# Severe Guillain-Barré syndrome after surgery for multiple fractures: a rare case report with a 5-year follow-up and a brief review of the literature

**DOI:** 10.1186/s12891-020-03864-4

**Published:** 2021-01-04

**Authors:** Jian Chen, Jian-xiong Ma, Cai-hong Zuo, Qing Zhang, Heng-ting Chen, Xin-long Ma

**Affiliations:** 1 Department of Orthopaedics, People’s Hospital of Xuancheng City, Xuancheng, China; 2 Institute of Orthopedics, Tianjin Hospital, Tianjin University, Tianjin, China

**Keywords:** Guillain-barré syndrome, Fracture, Postoperative paralysis, Respiratory failure, Rare case

## Abstract

**Background:**

Guillain-Barré syndrome (GBS) is the most common and serious acute paralytic neuropathy and is usually caused by infection. It is thought to be the result of an aberrant response of the immune system. To our knowledge, GBS, especially severe GBS, after orthopaedic surgery has rarely been reported.

**Case presentation:**

We herein report the case of a 58-year-old man who developed quadriplegia and respiratory failure on the 6th day after surgery for multiple fractures. The patient had no symptoms of respiratory or gastrointestinal tract infection within 4 weeks before the onset. The white blood cell count was normal, and there was no redness, swelling, heat or pain in the surgical incision. Brain, cervical and thoracic magnetic resonance imaging were normal, albuminocytological dissociation was found on cerebrospinal fluid examination, and electrophysiological examination showed that sensory and motor nerve evoked potentials could not be elicited. A diagnosis of post-traumatic GBS was made, and the patient was treated with intravenous immunoglobulin and plasma exchange, as well as supportive care and rehabilitation exercise. The length of stay was 18 months, and the in-hospital-related costs amounted to $127,171. At the last follow-up, the patient had recovered only grade 3 power in the upper limbs and grade 2 power in the lower limbs.

**Conclusions:**

Severe GBS is a rare complication after orthopaedic surgery. When progressive weakness occurs in trauma patients, the possibility of GBS should be considered, and cerebrospinal fluid and electrophysiological examinations should be performed in a timely manner. For patients with severe GBS after trauma, the treatment costs may be high, and the prognosis may be poor.

## Background

Guillain-Barré syndrome (GBS) is the most common and serious acute paralytic neuropathy worldwide [1]. At present, it is believed that its pathogenesis is related to abnormal autoimmunity [2], which mainly damages the spinal nerve roots and peripheral nerves [3, 4]. GBS is an uncommon autoimmune disorder characterized by progressive weakness and diminished deep tendon reflexes, affecting approximately 100,000 people worldwide each year [5]. There are very few reports of GBS, especially severe GBS, after orthopaedic surgery [6, 7]. The case presented here is the first to report the treatment process for severe GBS after multiple fractures, calculate the in-hospital related costs, and follow up with the patient for 5 years to analyse the prognosis and provide a reference for the clinical treatment of such patients in the future.

## Case presentation

**Fig. 1:**
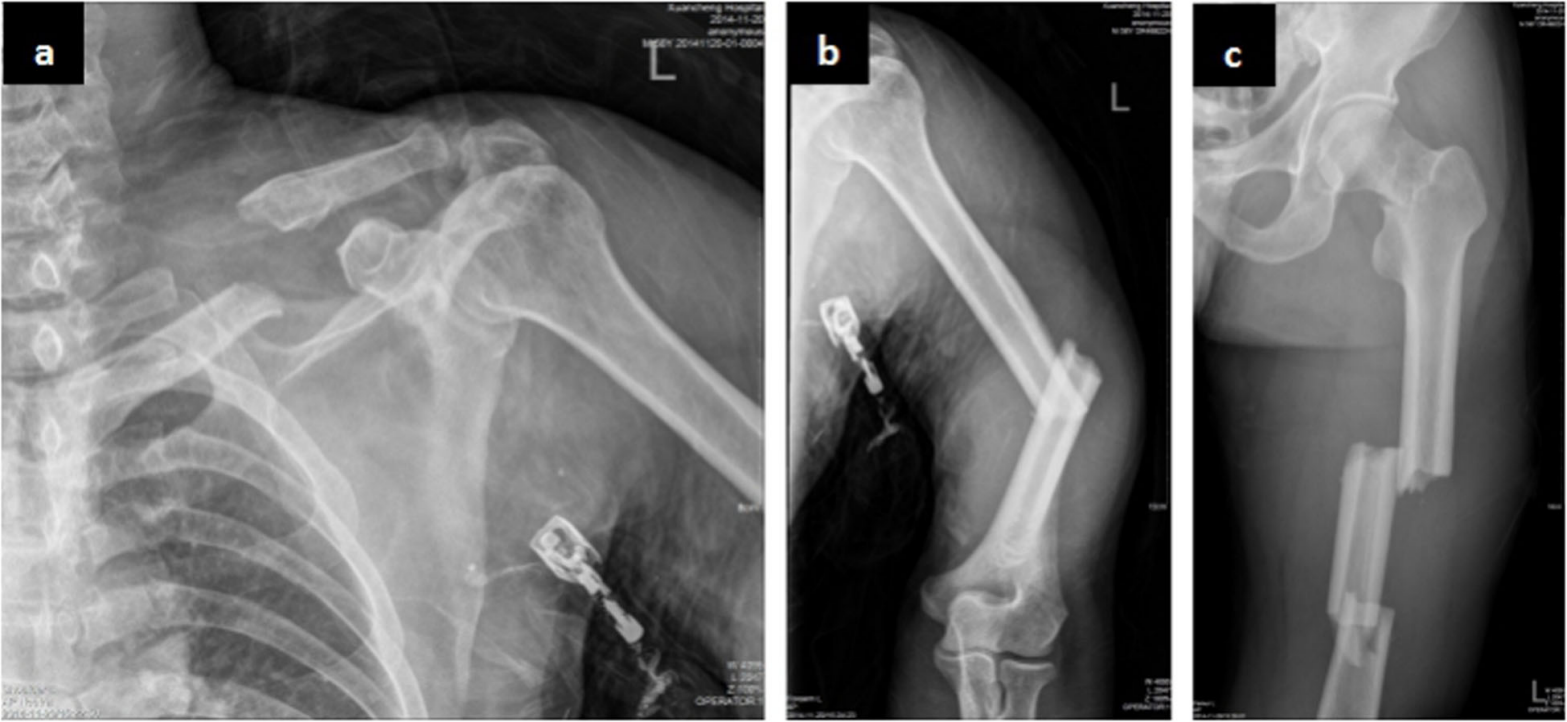
Radiographical assessment of multiple fracture, fractures of left clavicle (**a**), humeral shaft (**b**), femoral neck and femoral shaft (**c**)

**Fig. 2:**
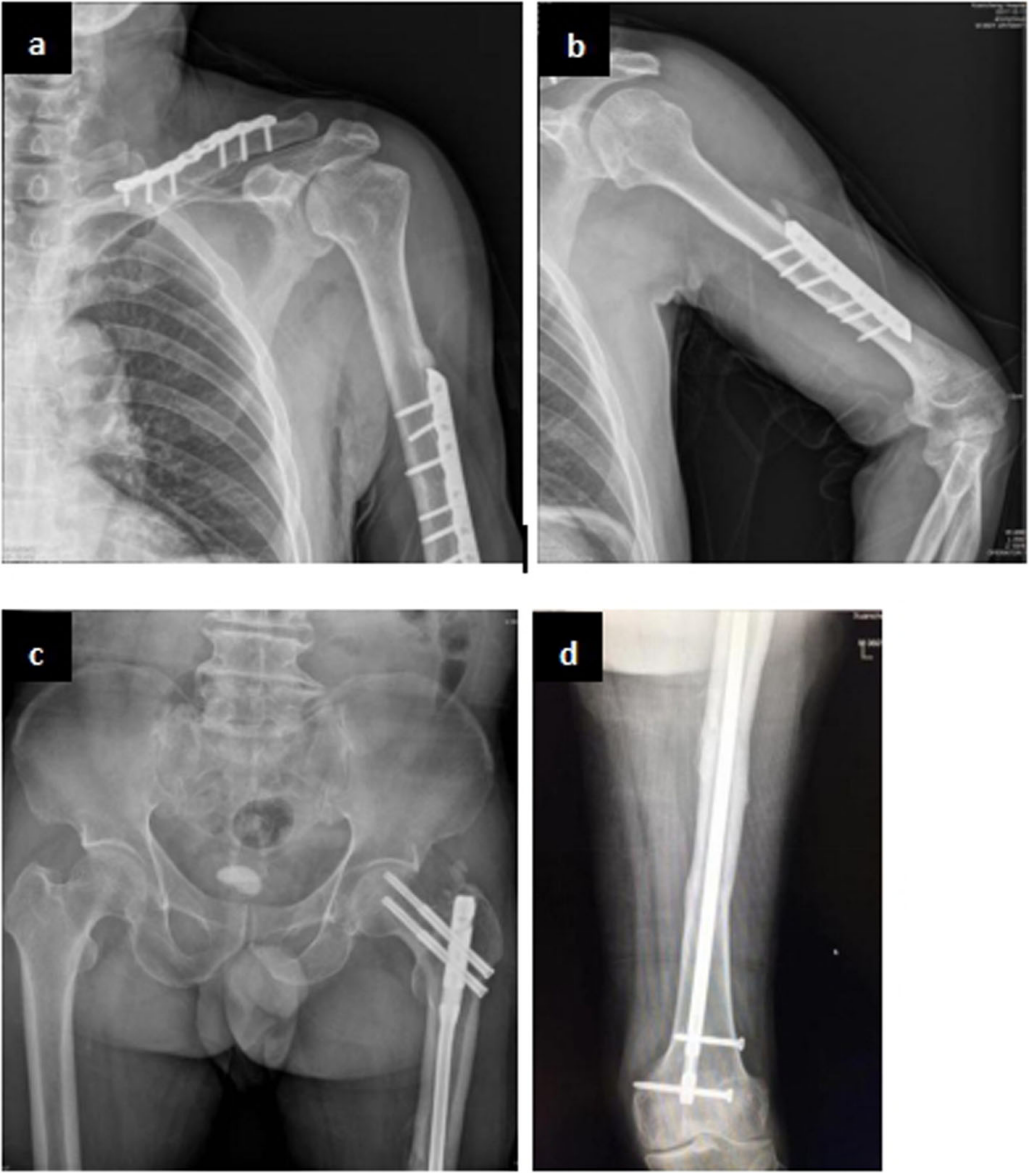
Postoperative radiograph of the left clavicle (**a**), humeral shaft (**b**), femoral neck and femoral shaft (**c**, **d**) (3 years after surgical)

After falling from a height of 5 m, a 58-year-old man was admitted to our hospital in November 2014 with fractures of the left clavicle, the humeral shaft, the left femoral neck, and the femoral shaft (Fig. 1). The left clavicle and humerus were fixed with steel plates, and the left femur was fixed with femoral reconstruction via an intramedullary nail (Fig. 2). During surgery for the humerus, we exposed the radial nerve and observed a contusion of the radial nerve with complete continuity.

**Fig. 3:**
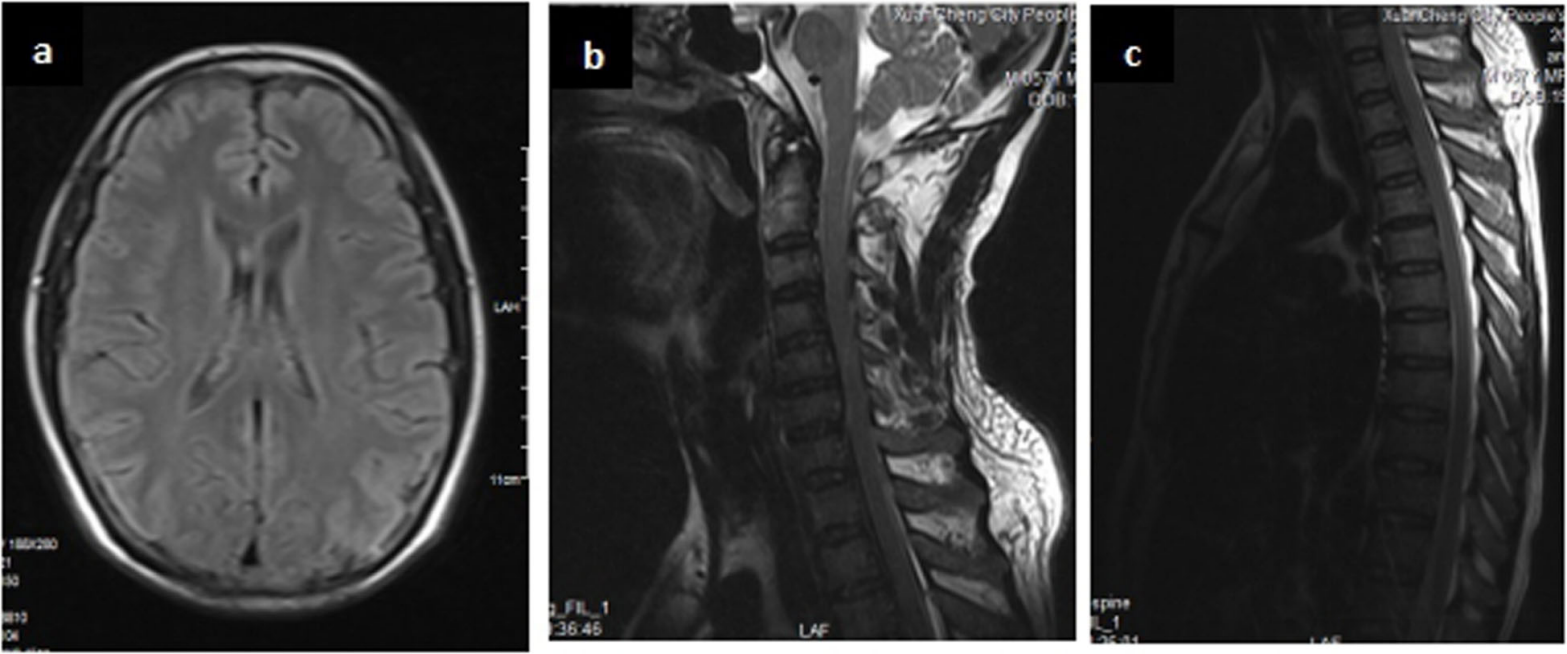
T2-weighted MRI showing normal Brain (**a**), cervical (**b**) and thoracic (**c**)

Six days after surgery, the patient developed progressive, symmetric Landry ascending weakness of his legs and arms, areflexia and sensorial disorder. Brain, cervical and thoracic magnetic resonance imaging (MRI) were normal (Fig. 3). The patient then developed acute respiratory failure, requiring immediate intubation and admission to the intensive care unit (ICU). The time from onset to admission to the ICU was 7 h. His axillary temperature was 36.7 °C, his pulse was 137 beats per min, his blood pressure was 168/95 mm Hg, his respiratory rate was 26 breaths per min, and his oxygen saturation was 78 percent. After mechanical ventilation, his respiratory failure symptoms improved, his pulse was 117 beats per min, his blood pressure was 138/90 mm Hg, his respiratory rate was 15 breaths per min, and his oxygen saturation was 100 percent. On the 1st day after admission to the ICU, the lumbar puncture was normal. On the 6th day, the lumbar puncture showed raised protein (1545 mg/L, normal < 450 mg/L) without pleocytosis. The cerebrospinal fluid (CSF) showed albuminocytological dissociation. Electrophysiological examination showed that sensory and motor nerve evoked potentials could not be elicited.

The clinical signs and CSF and electrophysiological examinations were in accordance with the diagnostic criteria of GBS. A diagnosis of post-traumatic Guillain-Barré syndrome (GBS) was made. In the Brighton classification [8], there was level 1 diagnostic certainty of GBS. High-dose intravenous immunoglobulin (0.4 g/kg of body weight per day for 5 days) was administered starting on the day of admission to the ICU. Because the clinical improvement was not significant, 3 plasma exchange treatments were subsequently started, as well as supportive care and rehabilitation exercise.

**Fig. 4:**
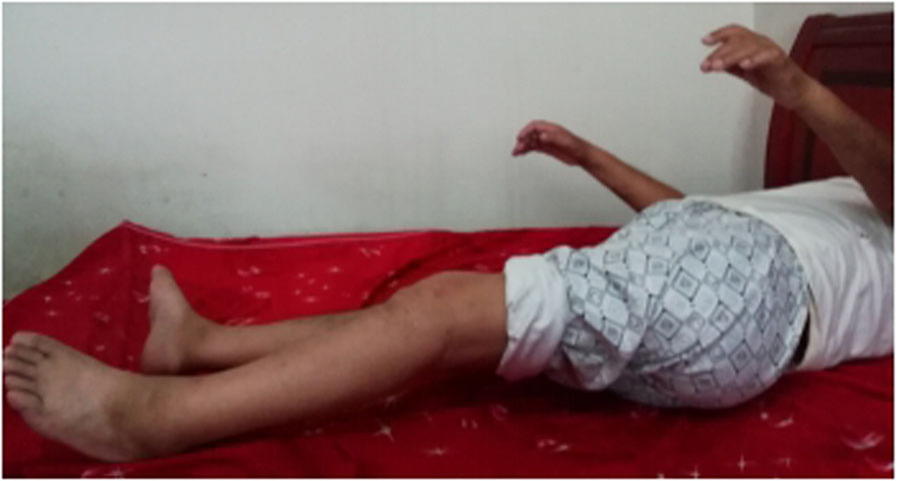
AT the last follow-up (5 years after surgery), grade 3 power in the upper limbs, grade 2 power in the lower limbs

A tracheotomy was performed on the 7th day after admission to the ICU. After 6 months, the patient’s respiration recovered, weaning from mechanical ventilation was started, and extubation was performed 9 months later. In mid-June 2015, our patient was transferred to the rehabilitation department for functional exercise. In June 2017, the patient was discharged from the hospital, and the power of both the upper and lower limbs was grade 2. In the hospital, the related costs for GBS treatment reached $127,171. At the last follow-up (5 years after surgery), however, the patient had recovered only grade 3 power in the upper limbs, grade 2 power in the lower limbs, and he had weakened deep tendon reflexes and impaired limb sensation (Fig. 4).

## Discussion and conclusion

As an immune-mediated disorder, GBS is usually caused by infection. Approximately two-thirds of patients have a history of respiratory or gastrointestinal tract infection, and at least one-third of these infections are caused by Campylobacter jejuni [9]. In addition, vaccination [10], influenza A virus [11] and Zika virus [12] can also cause GBS. It has also been reported to be associated with cardiac surgery and malignancy [13, 14]. However, GBS is rarely reported after trauma or orthopaedic surgery and is limited to case reports. In the case described by Lin et al. [15], GBS followed facial bone fracture, and the patient had a complete recovery 5 weeks later. The patient experienced progressive weakness in all 4 limbs, and all tendon reflexes were absent. Electrodiagnostic studies clearly revealed severe, generalized, predominantly motor demyelinating peripheral neuropathy. Hendawi and Zavatsky [6] documented a case of GBS after pelvic fracture fixation. They hypothesized that GBS was induced in this trauma patient by direct nerve injury caused by poorly positioned hardware and that the patient had a significant recovery in limb strength at last follow-up.

In our case, in addition to quadriplegia, the patient also had respiratory muscle paralysis. The purpose of acute treatment is to save lives. For patients with respiratory muscle paralysis, endotracheal intubation and ventilator-assisted respiration are given. After their condition is stabilized, the related immunotherapy and symptomatic treatment should be carried out. At present, intravenous immunoglobulin (IVIG) and plasma exchange have been proven to be effective methods in the treatment of GBS [16]. At the same time, physiotherapy is needed to promote the recovery of patient muscle strength and motor function. However, despite these treatments, some patients still have a severe disease course and residual disabilities. As demonstrated by our case, 5 years after surgery, the patient was still confined to bed or chair bound and met the level 4 diagnostic criteria of the GBS Disability Scale [17].

The physiopathology of GBS after trauma or orthopaedic surgery is still unclear. It may be related to immune stimulation caused by trauma or orthopaedic surgery, resulting in an aberrant autoimmune response to peripheral nerves and their spinal roots [18]. Clinical moter deficits and sensory deficits were obvious in our patient, electrodiagnostic examination revealed that motor and sensory nerve evoked potentials could not be elicited, supporting a diagnosis of the acute motor-sensory axonal neuropathy (AMSAN) variant of GBS.

In conclusion, our case highlights the possibility of GBS after multiple fractures. Because GBS is a life-threatening disease, it should be diagnosed as soon as possible in patients with rapid, progressive Landry ascending paralysis after trauma. If the diagnosis is in doubt, CSF and electrophysiological studies should be carried out in a timely manner to assist in GBS diagnosis. At the same time, interdisciplinary cooperation is needed to improve the level of clinical diagnosis and treatment and provide the best treatment for patients. Because the patient may have a severe disease course, poor outcome and high costs, it is necessary to communicate well with the patient’s family to avoid medical disputes.

## Data Availability

The authors declare that all data used during the study appear in the submitted article.

## Abbreviations

GBS: Guillain-Barré syndrome
MRI: Magnetic resonance imaging
ICU: Intensive-care unit
CSF: Cerebrospinal fluid
IVIG: Intravenous immunoglobulin
AMSAN: Acute motor-sensory axonal neuropathy
